# Retraction: Kinesin family member 3A stimulates cell proliferation, migration, and invasion of bladder cancer cells *in vitro* and *in vivo*

**DOI:** 10.1002/2211-5463.13756

**Published:** 2023-12-27

## Abstract

Kinesin family member 3A stimulates cell proliferation, migration, and invasion of bladder cancer cells *in vitro* and *in vivo*. Q. Zhou, J. Yu, Q. Zheng, T. Wu, Z. Ji, Y. Zhuo. *FEBS Open Bio* 11, 1487–1496.

https://doi.org/10.1002/2211-5463.12768.

The above article, published online on November 27, 2019, in Wiley Online Library (wileyonlinelibrary.com), has been retracted by agreement between the Editor‐in‐Chief Miguel A. De la Rosa, FEBS Press, and John Wiley and Sons Ltd. The retraction has been agreed following an investigation into concerns raised by a third party, which revealed inappropriate image duplications between this article and a previously published article [1]. Thus, the editors consider the conclusions of this manuscript substantially compromised.

[1] Kun Zuo, Youhong Zhao, Yukun Zheng, De Chen, Xiaoli Liu, Song Du & Qing Liu (2019) Long non‐coding RNA XIST promotes malignant behavior of epithelial ovarian cancer. *OncoTargets and Therapy*, 12: 7261–7267. DOI: 10.2147/OTT.S204369.

Kinesin family member 3A stimulates cell proliferation, migration, and invasion of bladder cancer cells *in vitro* and *in vivo*. Q. Zhou, J. Yu, Q. Zheng, T. Wu, Z. Ji, Y. Zhuo. *FEBS Open Bio* 11, 1487–1496.

https://doi.org/10.1002/2211-5463.12768.

The above article, published online on November 27, 2019, in Wiley Online Library (wileyonlinelibrary.com), has been retracted by agreement between the Editor‐in‐Chief Miguel A. De la Rosa, FEBS Press, and John Wiley and Sons Ltd. The retraction has been agreed following an investigation into concerns raised by a third party, which revealed inappropriate image duplications between this article and a previously published article [1]. Thus, the editors consider the conclusions of this manuscript substantially compromised.

[1] Kun Zuo, Youhong Zhao, Yukun Zheng, De Chen, Xiaoli Liu, Song Du & Qing Liu (2019) Long non‐coding RNA XIST promotes malignant behavior of epithelial ovarian cancer. *OncoTargets and Therapy*, 12: 7261–7267. DOI: 10.2147/OTT.S204369.

